# Access to translated invitations online increases involvement of linguistically diverse households in a population-based study: a cluster randomized controlled study

**DOI:** 10.1038/s41598-025-12092-6

**Published:** 2025-07-30

**Authors:** Paula S. Herrera-Espejel, Thomas Mildner, Hermann Pohlabeln, Christine Genedl, Lutz Jasker, Stefan Rach

**Affiliations:** 1 Department of Epidemiological Methods and Etiological Research, Leibniz Institute for Prevention Research and Epidemiology - BIPS, Achterstr. 30, 28359 Bremen, Germany; 2 Leibniz ScienceCampus Digital Public Health, Bremen, Germany; 3 Digital Media Lab, University of Bremen, Bremen, Germany; 4 Department of Biometry and Data Management, Leibniz Institute for Prevention Research and Epidemiology - BIPS, Bremen, Germany; 5 Institute for Quality Development in the State of Bremen (IQHB), Bremen, Germany

**Keywords:** Epidemiology, Recruitment, Multilingual, Nonresponse, Population-based, Public health, Epidemiology, Public health, Epidemiology

## Abstract

This double-blind cluster-randomized controlled study examined the effectiveness of a multilingual physical-digital recruitment strategy to increase the involvement of linguistically diverse households in the German sub-survey of the World Health Organization European Childhood Obesity Surveillance Initiative. Students in primary schools received German study invitations either alone or with a supplemental flyer containing a QR-code for accessing translations online. A total of 114 classrooms (2306 students) received the flyer, while 115 classrooms (2276 students) served as control group. Main outcomes were active of responses to invitations and study participation. Response proportions were 48% overall, 48.6% in the flyer and 47.4% in the control group. Participation proportions were 26.7% overall, 27.8% in the flyer and 25.4% (571/2251) in the control groups. The flyer increased the odds of participation (14.9, 95% CI 1.003–1.315), but not the odds of active responses. In medium-social-level schools, response and participation proportions in the flyer group rose by 1.5% (ROR 1.015, 95% CI 1.003–1.027) and by 2% (ROR 1.020, 95% CI 1.005–1.034), respectively, for each percentage point increase in the proportion of non-German speaking households per classroom. The flyer’s effect in low- and high-social level schools was inconclusive. Although modest in magnitude, the addition of the flyer increased overall participation and, in particular, the involvement of linguistically diverse households in medium-social-level schools.

**Supplementary Information:**

The online version contains supplementary material available at 10.1038/s41598-025-12092-6.

## Introduction

Culturally and linguistically diverse (CALD) individuals are often not offered the same opportunities to participate in public health or epidemiological (PHE) research in comparison to native-speakers of the official public language^1^. Their willingness to participate appears to vary depending on language capabilities, regardless of their equal eligibility and relevance to a study^2^. As a result, the characteristics of certain subgroups are not reflected in the data acquired for analysis, which in turn may limit the generalizability of research findings beyond native-speakers of a single language^3–5^.

The participation of CALD groups may be encouraged from the very onset of the research invitation process by ensuring linguistic inclusion as a basic element of information accessibility^6^. Two proven factors of invitation responsiveness under the control of researchers are the choice of language and the channel used for its communication^7–11^.

Being acquainted with language demographics and tailoring translations might, however, be challenging in certain data collection scenarios, such as in the case of population-based studies where samples are randomly drawn from public registry offices. In such scenarios, initial contact is often limited to postal recruitment modes, not easily allowing a multilingual outreach approach. First and foremost, researchers might at most only count with information on the individual’s name, postal address, gender, and citizenship, so that no specific information on household language is readily available to them. Although a person’s citizenship may be used to have a rough idea of their possible native language, citizenship does not provide fixed insights into their languages spoken at home, proficiency levels, nor preferences. Moreover, there might be a limited knowledge of the heterogeneity of the language demographics of the target population^12^, as well as no unitary data infrastructure available from which data on CALD subgroups may be specifically sampled^13^. Even then, with access to comprehensive population language statistics, providing multiple translations of PHE material may prove unfeasible or ineffective^14^. Researchers either face high translation costs or simply cannot access translations due to scarcity of services. In any case, the inclusion of multilingual versions of postal invitations could still prove impractical due to the physical limitations of both the paper-based envelopes and materials.

Strategies that take advantage of emerging technologies to reduce selection and non-response bias between targeted subgroups are a pertinent topic in PHE outreach and study recruitment^1^. With the widespread use of smartphones^15^ and increasing awareness of QR codes^16^, the incorporation of multiple translations via a digital mode as a supplement to physical invitation letters could be advantageous to increase linguistic inclusion and reduce possible underrepresentation of CALD subgroups in population-based studies, while also maintaining a convenient and affordable recruitment process^17^.

It is, however, important to also consider the diversity of socioeconomic backgrounds when designing and evaluating language sensitive inclusion strategies using digital means (e.g., QR codes). For instance, lower levels of income and education are generally associated with reduced engagement in PHE studies (e.g., clinical trials)^18^. Additionally, digital divides in access, affordability, literacy, and privacy concerns may also transcend language barriers^16,18^. Comprehensive evaluations need to consider these factors to obtain an in-depth understanding of both the effectiveness and inclusiveness of digitally-based approaches across different socioeconomic groups^19^.

### Objectives

This study is a cluster randomized experiment embedded in the German sub-survey within the World Health Organization (WHO) European Childhood Obesity Surveillance Initiative (COSI) in the Free Hanseatic City of Bremen. To explore the practical application of such a multimodal strategy, we designed a flyer in the form of a sticky note (henceforth, “the flyer”) with a QR-code, linking to a website containing multiple machine-translated versions of the original postal invitation information for the COSI study in German language. The primary objective of this strategy was to increase the overall participation of students in the COSI study. However, the multilingual component was aimed at their parents whose consent was required for their study participation. We hypothesized that providing CALD parents, who may have lower German proficiency, with access to translated study information would enable them to make informed decisions. Furthermore, effective parental involvement^20^, and linguistic inclusiveness^21^, specifically for CALD parents, have been demonstrated to encourage higher student participation in school activities. As such, using a multimodal strategy could result in a higher number of parental consents (i.e., a higher participation proportion), and, at the same time, in an increase in active refusals (as opposed to passive refusals, i.e., not responding). Together, these would lead to a higher number of valid returned consent forms. Therefore, we compared the likelihood of an active response to study invitations as well as eventual study participation among students in second and third grade classrooms who were randomized to receive either the regular invitation letter alone or in addition to a supplementary flyer attached on top of the letter.

## Methods

### Ethics approval

The German COSI sub-survey conducted in Bremen was approved by the ethics committee of the local chamber of physicians in Bremen, Germany (Bremen Medical Association, reference number 841) and the Senatorial Authority for Children and Education of the Free Hanseatic City of Bremen (reference number ID 2022-31). Written informed consent was obtained from all legal guardians (henceforth, parents) and, in addition, all children were asked for their assent prior to the examinations. The study was conducted in accordance with the Declaration of Helsinki of 1975 (in the current, revised version).

### Study design

This study is a double-blind, parallel, group cluster-randomized controlled experiment embedded into a survey following the WHO COSI protocol, jointly developed by the WHO Regional Office for Europe and participating Member States^22^. The aim of the study was to test whether a multilingual physical-digital PHE information strategy increases the active response of CALD groups to the COSI study invitation. The study was conducted based on the CONSORT 2010 statement and its extension for cluster randomized control trials^23^ and general guidelines for studies within a study regarding recruitment procedures were followed^24^.

COSI is a population-based surveillance initiative that provides standardized measurements of body weight and height for children aged six to nine years residing in more than 45 participating countries in the WHO European Region. Six rounds of data collection have been conducted regularly since 2007, with the most recent between 2021 and 2024. A detailed description of the study protocol can be found elsewhere^22^. In short, three record forms were used to collect COSI data. The mandatory child record gathers the children’s sex, age and anthropometric measurements (i.e., weight, height, hip and waist circumference). The mandatory school record form, completed by school staff, collects information about the number of children enrolled and measured per sampled classroom, as well as the characteristics of the school environment (e.g., number of students, availability of sports facilities and outside playground areas, availability of canteens and cafeterias). The optional family record form is completed by the children’s parents provides information on dietary and physical activity habits, screen time and sleep duration, as well as households characteristics, including questions on the main language spoken at home.

### Participants

Second and third grade students at 97 public primary schools in 15 school districts in Free Hanseatic City of Bremen, Germany, were eligible to participate in the COSI study. The COSI study protocol^22^ specifies that the number of measured children should be a minimum of 2800 children per age group. Based on the response of 40% obtained in COSI Round 5 in Bremen (2018/2019), it was estimated that 50 schools need to be recruited to obtain this goal. Using proportional sampling (based on the number of eligible students in a district), 50 schools were randomly selected from the school districts. If a school refused, another school was selected from the same district according to a random order already determined during the initial randomization. Personnel shortages on schools and field staff caused by the COVID-19 pandemic’s long-term impact severely delayed the recruitment and study measurements, forcing the study to be stopped early (due to the end of the school year) with only 35 schools enrolled, instead of the intended 50 schools. All second and third grade students (n = 4582) from these 35 schools were invited to participate in COSI.

### Recruitment

Principal’s offices were contacted by e-mail with the COSI study information, and notified that the field staff would contact them via phone. If there was no response up to seven further phone calls were attempted. During the phone call, principals or vice-principals were informed in detail about the study goals and procedures, and their consent was obtained for their school’s inclusion in the study.

Two appointments were arranged with the consenting schools. During the first one, the field staff handed out the pre-filled invitation envelopes to the teachers of eligible classrooms, instructing them on when and how to distribute the envelopes to their students, and how to collect the responses from parents. Teachers were instructed to collect the envelopes, store them unopened in a locked cabinet, and hand them to the field team on the examination day. During the second appointment, approximately four weeks later, the field team collected and reviewed returned consent forms and questionnaires, and invited all children with a valid parental consent to participate in the study’s anthropometric measurements. Completed questionnaires of children who were either absent or did not assent to participate in the measurements were excluded from the participant dataset.

Invitation materials consisted of sealed, opaque envelopes containing an invitation letter, a study information leaflet, two copies of a consent form, the family record form, and a return envelope. All documents were written in German. The invitation letter and leaflet informed parents about the COSI study in general, the anthropometric measurements that would be taken from their children, and that completing the questionnaire was not a prerequisite for their children’s participation in the anthropometric measurements. The consent form asked parents to either authorize or not the participation of their children in the COSI study, and to return the form, along with the questionnaire (if applicable), in the sealed return envelope via their children to their school.

### Treatment

**Fig. 1:**
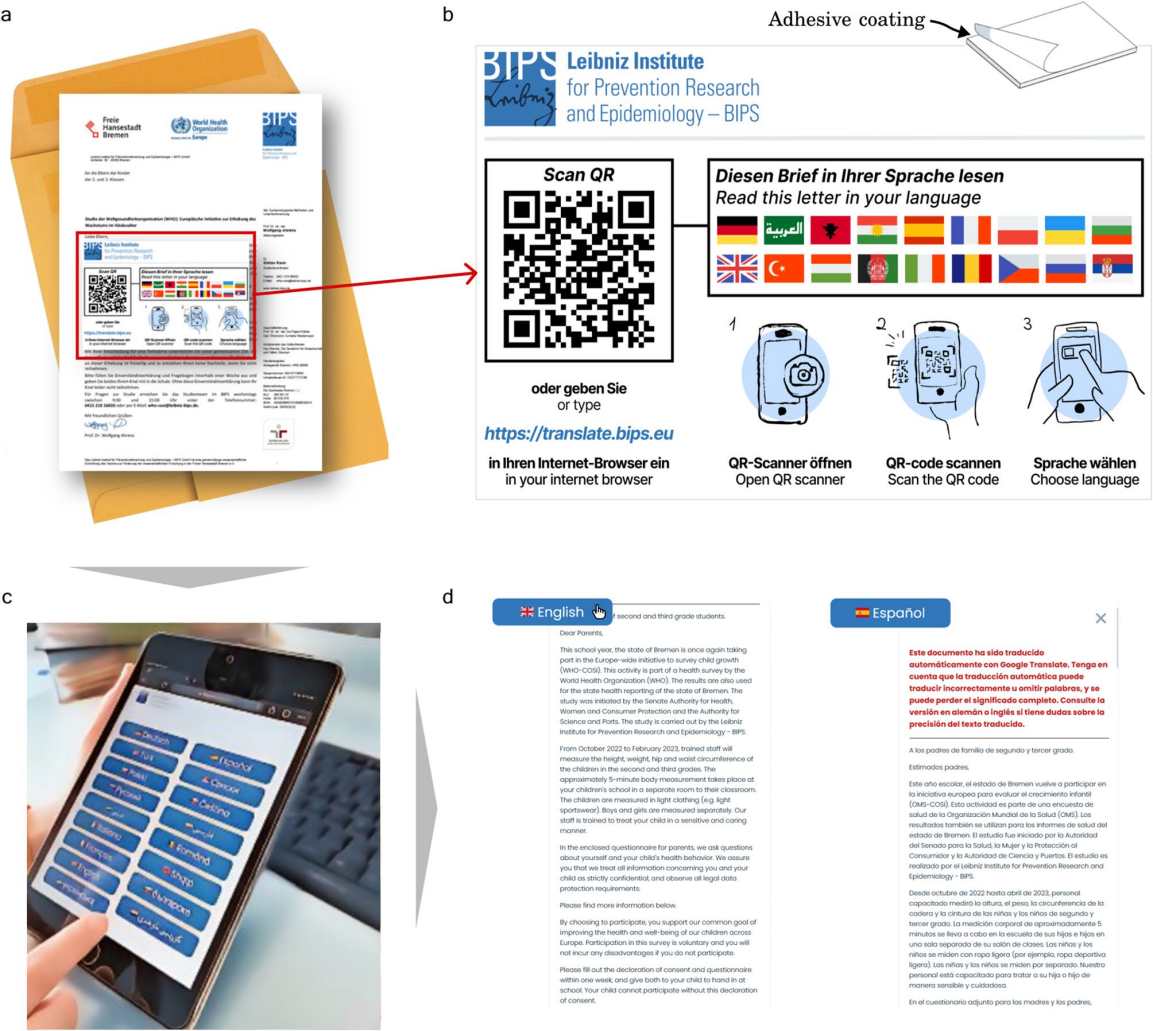
Visual methods summary of the user journey of the intervention (flyer with a QR code). (**a**) Original German paper version of the invitation letter version with adhesive flyer on top. (**b**) Magnification of the flyer with sticky-note-like adhesive coating on the back. (**c**) Mobile device with landing webpage for language selection. (**d**) Example of the English (left) and Spanish (right) translations texts (in black) with warning disclaimer about translation quality (in red).

A randomly selected subsample comprising approximately 50% of all classrooms was provided with access to translated study information via a flyer in the form of a sticky note attached to the top of the invitation in addition to the standard invitation material (Fig. 1a). The design of the flyer was informed by a co-design seminar with international graduate students. The visual content of the flyer consists of a QR code and its corresponding URL that links to a website with multiple machine-translated versions of the original German study information, several national flag icons reflecting available language versions, and non-verbal instructions in form of pictograms on how to use the QR code with a smartphone (Fig. 1b). The website consisted of a landing page with buttons for language selection (Fig. 1c), which opened a text window containing translated versions of the invitation letter and the study information (Fig. 1d). Available languages included German, English and 15 machine-translated language versions (Albanian, Arabic, Bulgarian, Czech, French, Italian, Kurdish, Persian, Polish, Romanian, Russian, Serbian, Spanish, Turkish and Ukrainian). Languages were selected based on migration statistics of the Free Hanseatic City of Bremen from the Federal Office of Statistics in Germany as of November 2022^25^.

All machine-translated texts were accompanied by a disclaimer message (Fig. 1d) in red fonts, warning its readers that the texts had been machine-translated and could therefore contain errors, and that accurate translations were available in German and English. The website was deliberately kept simple to facilitate its use and compatibility across different web browsers.

The English version was translated from German by the authors (PSHE, SR). All other translations were created based on the English text source using the freely-available online MT service Google Translate. The translations were validated by speakers fluent in each language using a short quality-assessment questionnaire (see Supplementary Questionnaire S1). The texts with accuracy errors were then edited accordingly^17^. One language (Hungarian) was excluded from the website because the post-editing exercise indicated that the quality of the machine translation from English was insufficient and did not preserve meaning enough to be used.

### Randomization and masking

In each grade of each participating school, enrolled classrooms were randomly assigned to either the flyer or control group in a 1:1 ratio. Since the invitation materials were sealed in opaque envelopes, and it was not disclosed that some envelopes contained an additional flyer, students, parents, and teachers handing out the envelopes were blinded to the group allocation. The field team responsible for recruitment and measurements, the counter of the returned consent forms (PSHE), and the data entry personnel were unaware of the group allocation while performing these tasks. Throughout the data preprocessing and analysis, PSHE and HP had no access to the group allocation until the final step of the data analysis (i.e., the modelling). SR, who independently performed all analyses in parallel for validation purposes, had access to the group allocation during all research steps, but did not use it until the modelling.

Sequence generation for random selection of schools and group allocation was performed by SR using the statistical software R (version 4.1.2) with the “sampling” package^24^. SR had had no active role in recruitment and examinations, aside from supervisory functions.

### Outcomes and explanatory variables

Outcomes of interest were the contact proportion^26^, measured as the percentage of invited children who returned the consent form regardless of the validity of the response (henceforth, “active response”), and the response proportion^26^, determined as the percentage of invited children eventually participating in the COSI examinations.

The main explanatory variable was the assignment to the experimental group (flyer vs. control). Data on school demographics provided by Institute for Quality Development in the State of Bremen (IQHB) were used to quantify the linguistic diversity of classrooms and socioeconomic status of schools. Household language proficiency was quantified by the average percentage of students per grade from families where German is not the main language spoken at home (i.e., “non-German households”). For descriptive analyses shown in Fig. 3, the percentage of non-German households was stratified into three groups ([0–33], [34–66], [37–100]). The school’s social level (SSL) was classified as being either low, medium, or high based on an index of small-scale socioeconomic characteristics of schools’ catchment areas, as well as the proportion of students requiring special language training, special educational needs, and additional financial assistance averaged over previous years^27^. Additional variables to control for confounding were the number of students per school and per classroom, the proportion of female students per classroom, and the students’ grade (either second or third), all as reported in the school record form. Variables were grand mean centered where appropriate.

In a third analysis, we examined whether there were differences in the composition of the groups that received the flyer as compared to the control group, using the country of birth of the child and parents, and the language predominantly spoken at home as reported on the family record form.

### Statistical analysis

For the first and second analyses, multilevel mixed-effects logistic modeling techniques were used to estimate odds ratios (OR) and 95% confidence intervals (CI)^28^. The models were constructed assuming that the likelihood of an active response (Model 1) or eventual study participation in the measurements (Model 2) depended on language proficiency (i.e., the percentage of non-German households), and that a potential effect of the flyer would moderate this association. Both models included the variables group (flyer vs. control), the percentage of non-German households (grand mean centered), and SSL (dummy coded with reference category “medium”), as well as variables to control for confounding as listed above. To account for the cluster sampling design, the multilevel model utilized schools nested in school districts as second and third level variables to estimate random intercepts.

Average treatment effects (ATE) based on G-computation^29^ were calculated by comparing counterfactual predictions using the R package *emmeans*^30^.

Because the percentage of non-German households might vary across different SSL, two further models, Models 1a and 2a included a three-way interaction between group (flyer vs. control), the percentage of non-German households (grand mean centered), and SSL (dummy coded with reference category “medium”), as well as, all corresponding lower two-way interactions in addition to all main effects. An additional file provides supplementary methods regarding the three-way interaction in the multilevel logistic regression (see Supplementary Equation S1). Since in logistic regression modeling estimated coefficients for interaction effects do not translate into OR but rather into ratios of OR (ROR)^31^, the correct OR were recalculated using the R package *emmeans*^30^ to obtain the effect of the flyer in the three-way interaction for each SSL.

To account for the sampling design and nonresponse among schools, survey weights were calculated in a two-step procedure^32^. First, to correct for unequal inclusion probabilities of schools due to the sampling design of the study, design weights were calculated using the Horvitz-Thompson-Estimator^33^, defined as the inverse of the inclusion probability. As a second step, calibration weights were calculated for each grade in each school by iterative proportional fitting (“raking”)^34^ using the joint distribution of SSL (low, medium, high) and the percentage of non-German households stratified into three groups ([0–33], [34–66], [37–100]), as well as the marginal distribution of the number of students per grade stratified into four groups ([< = 50], [51–60], [61–70], [71–80], [> 80]). Survey weights were obtained by multiplying design and calibration weights and trimming them to the 1st and 99th percentile to lower the variance of the weights and reduce the influence of outliers.

For the third analysis, we used the responses from the completed questionnaires to compare the student’s characteristics, including their anthropometric measurements and the social features of their household. We performed chi-square tests (categorial variables) and t-tests (continuous variables) to screen for systematic differences.

All analyses were conducted with the statistical software R, version 4.3.2. Models were estimated with the *lme4* package^35^, while standardized mean differences^36^ and 95% CI were calculated using packages *gtsummary*^37^ and *smd*^38^.

### Role of the funding source

The funder of the study had no role in study design, data collection, data analysis, data interpretation, or writing of the report.

## Results

**Fig. 2:**
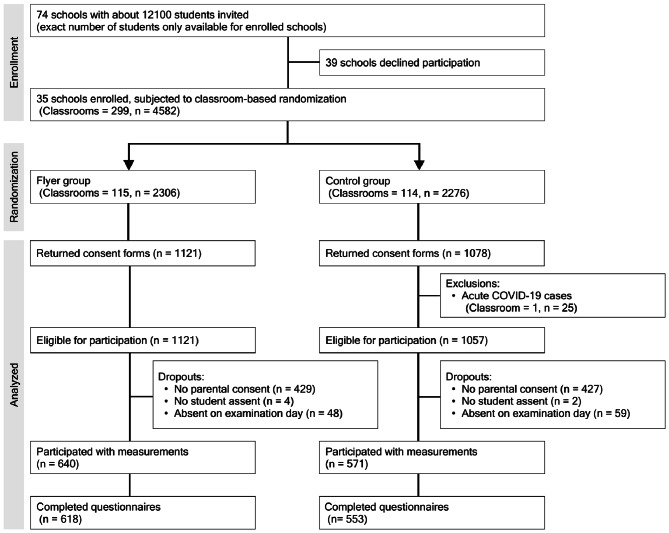
CONSORT Flowchart of the cluster randomized controlled study.

Of the 74 schools invited, 35 agreed to participate in the COSI study with 4582 students from 229 classrooms randomized to either the flyer or control group (Fig. 2, Table 1). A total of 115 classrooms with 2306 students (51%) received the invitation materials along with the flyer, while 114 classrooms with 2276 students (49%) served as the control group.

**Table 1:** Descriptive analysis of the study sample.

Variables	Invited households (N = 4582)				Eligible participants (N = 4557)			
	Control group N = 2276	Flyer group N = 2306	SMD^a^	(95% CI^b^)	Control group N = 2251	Flyer group N = 2306	SMD^a^	(95% CI^b^)
Percentage of non-German households^c^	37.9 (22.3)	39.2 (21.4)	− 0.06	(− 0.11, 0.00)	38.3 (22.1)	39.2 (21.4)	− 0.04	(− 0.10, 0.02)
Percentage of non-German households			0.05	(− 0.01, 0.11)			0.04	(− 0.02, 0.10)
[0–33]	1077 (47.3%)	1043 (45.2%)			1052 (46.7%)	1043 (45.2%)		
[34–66]	844 (37.1%)	906 (39.3%)			844 (37.5%)	906 (39.3%)		
[37–100]	355 (15.6%)	357 (15.5%)			355 (15.8%)	357 (15.5%)		
School Social Level (SSL)			0.05	(− 0.01, 0.10)			0.03	(− 0.03, 0.09)
Low	625 (27.5%)	663 (28.8%)			625 (27.8%)	663 (28.8%)		
Medium	931 (40.9%)	961 (41.7%)			931 (41.4%)	961 (41.7%)		
High	720 (31.6%)	682 (29.6%)			695 (30.9%)	682 (29.6%)		
Sex			0.02	(− 0.04, 0.07)			0.02	(− 0.04, 0.08)
Female	1120 (49.2%)	1116 (48.4%)			1110 (49.3%)	1116 (48.4%)		
Male	1156 (51.8%)	1190 (52.6%)			1141 (51.7%)	1190 (52.6%)		
School Grade			0.04	(− 0.02, 0.09)			0.02	(− 0.03, 0.08)
Second	1184 (52.0%)	1159 (50.3%)			1159 (51.5%)	1159 (50.3%)		
Third	1092 (48.0%)	1147 (49.7%)			1092 (48.5%)	1147 (49.7%)		
Classroom Size^c^	20.9 (3.61)	20.9 (3.45)	− 0.01	(− 0.07, 0.04)	20.8 (3.60)	20.9 (3.45)	− 0.03	(− 0.09, 0.03)
School Size^c^	139.4 (35.9)	141.1 (36.1)	− 0.05	(− 0.10, 0.01)	139.9 (35.9)	141.1 (36.1)	− 0.03	(− 0.09, 0.02)

^a^Standardized mean difference.

^b^CI, Confidence Interval.

^c^Mean (SD); n (%).

### Impact of the flyer on active responses

**Fig. 3:**
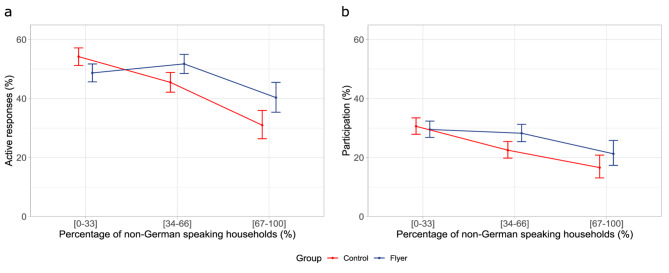
Observed effect of having access to online translations as a function of the percentage of non-German speaking households in the classroom. Mean percentages and 95% CIs are displayed for the flyer (blue) and the control group (red) across different levels of non-German speaking households ([0–33%], [34–66%], [37–100%]). (**a**) Active responses to study invitation (defined as returning the consent form). (**b**) Study participation.

The primary analysis focused on the effect of the flyer on the odds of an active response. A total of 2199 invited students responded actively resulting in an overall contact proportion of 48% (2199/4582), 48.6% (1121/2306) in the flyer group, and 47.4% (1078/2276) in the control group. Stratifying the sample with regard to the percentage of non-German speaking households into three groups ([0–33], [34–66], [37–100]) revealed that the difference in active responses between flyer and control increased with the percentage of non-German speaking households (Fig. 3a).

A multilevel mixed-effects logistic regression (Table 2, Model 1), however, revealed that the flyer did not increase the odds of active responses (OR 1.081, 95% CI 0.957**–**1.220). The odds of active responses were significantly lower for students in low-SSL compared to those in medium levels (OR 0.49, 95% CI 0.337**–**0.725). Correspondingly, the ATE of adding the flyer amounted to an increase of 1.85 percentage point, which, however, did not differ systematically from zero (1.85, 95% CI − 1.05 to 4.74).

**Table 2:** Mixed effects logistic regression model with interactions for outcome “active response of household to study invitation” (i.e., returned consent forms) for all invited students (n = 4582) and “Study Participation” for all eligible students (n = 4557).

	Model 1: Active response of invited households (n = 4582)				Model 2: Study participation of eligible participants (n = 4557)			
	No (N = 2383) n (%)	Yes (N = 2199) n (%)	OR	95% CI	No (N = 3346) n (%)	Yes (N = 1211) n (%)	OR	95% CI
**Main variables**								
Flyer								
No (Control)	1198 (50.3)	1078 (49.0)	1	reference	1680 (50.2)	571 (47.2)	1	reference
Yes	1185 (49.7)	1121 (51.0)	1.081	0.957–1.220	1666 (49.8)	640 (52.8)	**1.149**	**1.003**–**1.315**
Non-German households								
Mean (SD)	2.40 (22.2)	− 2.60 (21.1)	0.999	0.992–1.006	1.33 (21.8)	− 3.67 (21.1)	1.001	0.994–1.009
SSL								
Low	848 (35.6)	440 (20.0)	**0.494**	**0.337**–**0.725**	1062 (31.7)	226 (18.7)	**0.617**	**0.437**–**0.872**
Medium	952 (39.9)	940 (42.7)	1	reference	1408 (42.1)	484 (40.0)	1	Reference
High	583 (24.5)	819 (37.2)	1.273	0.847–1.912	876 (26.2)	501 (41.4)	**1.701**	**1.178**–**2.457**
**Covariates**								
School grade								
Second	1202 (50.4)	1141 (51.9)	1	reference	1693 (50.6)	625 (51.6)	1	reference
Third	1181 (49.6)	1058 (48.1)	0.924	0.819–1.044	1653 (49.4)	586 (48.4)	0.937	0.818–1.072
Classroom size								
Mean (SD)	− 0.255 (3.54)	0.277 (3.49)	1.030	0.995–1.066	− 0.119 (3.57)	0.329 (3.36)	1.006	0.970–1.043
Percentage of female								
Mean (SD)	− 0.350 (8.27)	0.379 (8.07)	1.003	0.994–1.012	− 0.103 (8.22)	0.285 (8.06)	0.999	0.989–1.009
School size								
Mean (SD)	1.55 (36.3)	− 1.68 (35.6)	0.997	0.992–1.001	0.609 (36.1)	− 1.68 (35.6)	0.999	0.995–1.003

Because the percentage of non-German households may vary across different socio-economic school environments, a second multilevel mixed-effects logistic regression accounted for the interplay between the frequency of non-German speaking households and socio-economic school environment (henceforth: “social school level”, SSL). This analysis provided evidence that the addition of the flyer providing access to multilingual translations affected the association between the percentage of non-German households and the odds of an active response (Table 3, Model 1).

**Table 3:** Full mixed effects logistic regression models for outcome “active response of household to study invitation” (i.e., returned consent forms) for all invited households (n = 4582) and “study participation” for all eligible students (n = 4557) (incl. covariates).

	Model 1a: Active response of invited households (n = 4582)				Model 2a: Study participation of eligible participants (n = 4557)			
	No (N = 2383) n (%)	Yes (N = 2199) n (%)	OR	95% CI	No (N = 3346) n (%)	Yes (N = 1211) n (%)	OR	95% CI
**Main variables**								
Flyer								
No (Control)	1198 (50.3)	1078 (49.0)	1	Reference	1680 (50.2)	571 (47.2)	1	Reference
Yes	1185 (49.7)	1121 (51.0)	1.030	0.853–1.243	1666 (49.8)	640 (52.8)	1.010	0.814–1.253
Non-German households								
Mean (SD)^b^	2.40 (22.2)	− 2.60 (21.1)	0.995	0.983–1.007	1.33 (21.8)	− 3.67 (21.1)	0.996	0.984–1.009
SSL								
Low	848 (35.6)	440 (20.0)	**0.445**	**0.284**–**0.698**	1062 (31.7)	226 (18.7)	**0.508**	**0.323**–**0.797**
Medium	952 (39.9)	940 (42.7)	1	Reference	1408 (42.1)	484 (40.0)	1	Reference
High	583 (24.5)	819 (37.2)	1.014	0.554–1.859	876 (26.2%)	501 (41.4)	1.419	0.790–2.547
**Two-way interactions**								
Flyer x Non-German households								
Mean (SD)	1.88 (21.8)	− 0.747 (20.8)	**1.015**	**1.003**–**1.027**	1.45 (21.5)	− 2.31 (21.0)	**1.020**	**1.005**–**1.034**
Flyer x SSL								
Low	416 (35.1)	247 (22.0)	1.204	0.823–1.760	533 (32.0)	130 (20.3)	1.451	0.910–2.314
Medium	476 (40.2)	485 (43.3)	1	Reference	708 (42.5)	253 (39.5)	1	Reference
High	293 (24.7)	389 (34.7)	1.072	0.605–1.899	425 (25.5)	257 (40.2)	0.954	0.526–1.732
SSL x Non − German households								
Low	18.7 (21.4)	19.2 (18.9)	1.003	0.985–1.021	18.5 (20.9)	19.5 (19.0)	1.007	0.988–1.027
Medium	1.44 (15.7)	3.49 (15.2)	1	Reference	1.80 (15.6)	3.59 (15.1)	1	Reference
High	− 19.8 (8.41)	− 21.3 (9.66)	0.987	0.960–1.015	− 20.3 (8.61)	− 21.1 (9.68)	0.995	0.970–1.022
**Three-way interaction**								
Flyer x SSL x Non-German households								
Low	18.1 (21.7)	19.3 (19.0)	0.990	0.973–1.007	18.2 (21.0)	18.7 (19.5)	**0.977**	**0.957**–**0.997**
Medium	0.679 (15.3)	4.68 (14.4)	1	Reference	1.41 (15.2)	5.52 (13.9)	1	Reference
High	− 19.2 (8.20)	− 20.2 (9.56)	0.994	0.967–1.022	− 19.6 (8.54)	− 20.6 (9.71)	0.973	0.946–1.001
**Covariates**								
School grade								
Second	1202 (50.4)	1141 (51.9)	1	Reference	1693 (50.6)	625 (51.6)	1	Reference
Third	1181 (49.6)	1058 (48.1)	0.940	0.832–1.062	1653 (49.4)	586 (48.4)	0.945	0.824–1.083
Classroom size								
Mean (SD)^b^	− 0.255 (3.54)	0.277 (3.49)	1.020	0.984–1.058	− 0.119 (3.57)	0.329 (3.36)	0.996	0.960–1.034
Percentage of female								
Mean (SD)^b^	− 0.350 (8.27)	0.379 (8.07)	1.003	0.994–1.012	− 0.103 (8.22)	0.285 (8.06)	0.999	0.989–1.009
School size								
Mean (SD)^b^	1.55 (36.3)	− 1.68 (35.6)	0.997	0.993–1.002	0.609 (36.1)	− 1.68 (35.6)	0.999	0.995–1.004

**Fig. 4:**
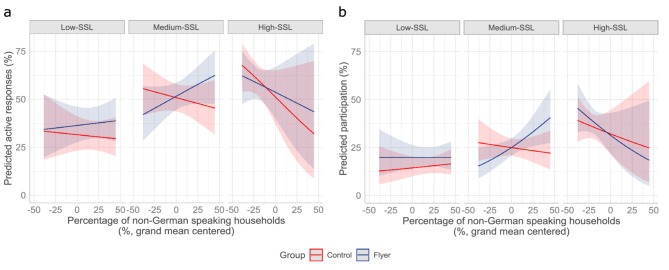
Predicted effect of having access to online translations as a function of school social level and the percentage of non-German speaking households in the classroom. Predicted percentages and 95% CIs are displayed for the flyer (blue) and the control group (red) across different social school levels (low, medium vs. high) and the percentage of non-German speaking households per classroom (**a**) Predicted active responses to study invitation (defined as returning the consent form). (**b**) Predicted study participation.

For each percentage point increase of non-German households in a classroom in a medium-SSL, the ratio of odds ratio (ROR) between control and flyer group increased by 1.5% (ROR 1.015, 95% confidence interval (CI) 1.003–1.027). Specifically, in the control group, each percentage point increase in non-German households decreased the odds ratio (OR) of actively responding by 0.05% (OR 0.995, 95% CI 0.983–1.007), while these odds increased by 1% (OR 1.010, 95% CI 0.997–1.023) in the flyer group. For classrooms in low- and high-SSL, the observed differences between control and flyer group were inconclusive as the CIs included 1 for both ROR. The increase in the ROR per percentage point increase in non-German households was 0.4% (ROR 1.004, 95% CI 0.992–1.017) and 0.9% (ROR 1.009, 95% CI 0.984–1.034) for classrooms in low- and high-SSL, respectively (see Table 4, left column, for the corresponding ORs). The visual representation of the three-way interaction effects predicted by the model is shown in Fig. 4a. Only in the control group, the odds of active responses were significantly lower in classrooms from low-SSL as compared to those in medium-SSL (OR 0.445, 95% CI 0.284–0.698) (Table 3, Model 1a).

**Table 4:** Estimated marginal means for the three-way interaction for the outcome’s active response (Model 1, left half of the table) and study participation (Model 2, right half of the table).

	Active responses			Study participation		
	Low SSL^a^	Medium SSL	High SSL	Low SSL	Medium SSL	High SSL
Control Group^b^	0.998 (0.985–1.011)	0.995 (0.983–1.007)	0.982 (0.958–1.007)	1.004 (0.989–1.018)	0.996 (0.984–1.009)	0.992 (0.969–1.015)
Flyer Group^b^	1.002 (0.990–1.015)	1.010 (0.997–1.023)	0.991 (0.967–1.016)	1.000 (0.987–1.013)	1.016 (1.002–1.030)	0.984 (0.962–1.007)
Ratio of ORs^c^Flyer/control	1.004 (0.992–1.017)	1.015 (1.003–1.027)	1.009 (0.984–1.034)	0.996 (0.982–1.011)	1.020 (1.005–1.034)	0.992 (0.968–1.017)

^a^SSL: School social level.

^b^ORs and 95% CI for the three-way interaction between group, SSL and the percentage of non-German speaking households.

^c^Ratio between the OR in the flyer and in the control group and 95% CI.

### Impact of the flyer on study participation

The second analysis investigated whether adding the flyer influenced the eventual study participation. This analysis used data from 4557 students, 25 students less than in the first analysis (Fig. 2). These students were enrolled in a classroom in the control group which was excluded from participating in the measurements at the day of examination due to COVID-19 cases present in their classroom. Out of the 4557 students, 1322 provided a valid informed consent form agreeing to participate in the measurements. However, 105 students were absent from the school on the day of the measurements, and 6, although present, did not assent to participate, leaving a total of 1211 students to be measured. This resulted in an overall response proportion of 26.7% (1211/4557), 27.8% (640/2306) in the flyer group, and 25.4% (571/2251) in the control group. Stratifying the sample with regard to the percentage of non-German speaking households into three groups revealed that also the difference in study participation between flyer and control group increased with the percentage of non-German speaking households (Fig. 3b).

A multilevel mixed-effects logistic regression (Table 2, Model 2) revealed that the odds of participating in the study were 14.9% higher for students who had received the flyer (OR 1.149, 95% CI 1.003–1.315). The odds also increased if students were enrolled in a high-SSL classroom (OR 1.70, 95% CI 1.178–2.457), and decreased for students in a low-SSL classroom (OR 0.62, 95% CI 0.437–0.872). Correspondingly, the ATE of adding the flyer amounted to an increase of 2.6 percentage points in participation (2.61, 95% CI 0.06–5.16) .

A regression additionally accounting for the interplay of the frequency of non-German speaking households and SSL indicated that adding the flyer improved study participation in classrooms in medium-SSL (Tables 3, Model 2a; Table 4, right column). For each percentage point increase of non-German households in these classrooms, the ROR between control and flyer group increased by two percent per percentage point increase in non-German households (ROR 1.020, 95% CI 1.005–1.034). In the control group, the odds of participating decreased by 0.04% (OR 0.996, 95% CI 0.984–1.009) per percentage point increase in non-German households, while in the flyer group the odds of participation increased by 1.6% (OR 1.016, 95% CI 1.002–1.030). Similar effects could not be observed for classrooms in low- and high- SSL. The decrease in ROR per percentage point increase of non-German households was 0.4% (ROR 0.996, 95% CI 0.982–1.011) and 0.8% (ROR 0.992, 95% CI 0.968–1.017) for classrooms in low- and high-SSL, respectively (Fig. 4b; see Table 4, right column, for the corresponding ORs). It was also observed that only in the control group, the odds of participation were significantly lower in low-SSL classrooms as compared to medium-SSL classrooms (OR 0.507, 95% CI 0.323–0.797) (Table 3, Model 2a).

### Weighted analyses

Given the response proportion among schools was only 47.3% (35 / 74 schools), all analyses were repeated with survey weights that account for the sampling design and nonresponse (Supplementary Tables S2 and S4). None of the results outlined above did change markedly in the weighted analyses, with the notable exception of an increased main effect of the flyer on the odds of an active response to 1.147 (95% CI 1.015–1.297) (Supplementary Table S2, Model 1), which translated into an increased ATE of adding the flyer of 3.24 percentage points (3.24, 95% CI 0.04–6.12) (Supplementary Table S3).

### Impact of the flyer on the sample composition

The third analysis assessed whether the flyer influenced the composition of students who participated with their anthropometric measurements and returned completed questionnaire.

Out of the 1211 participating students, a total of 1171 returned a completed questionnaire, resulting in an overall response proportion of 25.7% (1171/4557), 26.8% (618/2306) in the flyer group, and 24.6% (553/2251) in the control group (Table 5). Of the students who returned the questionnaire, over 80% were born in Germany (n = 959). About two-thirds of them speak native German at home with their parents (n = 681), while one fifth speak German as a second language (n = 241), and another fifth does not use German as a household language (n = 235). More than half of the students have parents who were both born in Germany (n = 608), more than a third have parents who were both born abroad (n = 366), and about 14% have one parent born in Germany and one born abroad (n = 160). No evidence was found that this pattern differed significantly between the flyer and the control group.

**Table 5:** Influence of the flyer on the sample composition.

Anthropometric measurements (N = 1211)	Control group	Flyer group	Chi-squared- and t-tests
	(N = 571)	(N = 640)	
Sex			
Male Female	284 (49.7%) 287 (50.3%)	314 (49.1%) 326 (50.9%)	X^2^(1, N = 1211) = 0.03129, *p* = 0.8596
Age in years^a^			
Mean (SD) Body Mass Index^b^	8.67 (0.621)	8.70 (0.645)	t(1202.1) = − 0.68308, *p* = 0.4947
Mean (SD)	16.95 (2.88)	17.03 (2.84)	t(1184.8) = − 0.45978
Missing	3 (0.5%)	1 (0.2%)	*p* = 0.6458
Waist-Hip Ratio^c^			
Mean (SD) Missing	0.836 (0.0523) 10 (1.8%)	0.834 (0.0482) 11 (1.7%)	t(1143.9) = 0.5975, *p* = 0.5503

Questionnaire data (N = 1171)	(N = 553)	(N = 618)	
Language spoken at home			
Native German household German as a second language	329 (59.5%) 109 (19.7%)	352 (57.0%) 132 (21.4%)	X^2^(3, N = 1171) = 0.90274, *p* = 0.8248
No German spoken at home	108 (19.5%)	127 (20.6%)	
Missing	7 (1.3%)	7 (1.1%)	
Country of birth of the student			
Student born in Germany Student born outside Germany	456 (82.5%) 94 (17.0%)	503 (81.4%) 111 (18.0%)	X^2^(2, N = 1171) = 0.24879, *p* = 0.883
Missing	3 (0.5%)	4 (0.6%)	
Country of birth of parents			
Both parents born in Germany One parent born in Germany	290 (52.4%) 80 (14.5%)	318 (51.5%) 80 (12.9%)	X^2^(3, N = 1171) = 2.3034, *p* = 0.5119
Both parents born outside Germany	163 (29.5%)	203 (32.8%)	
Missing	20 (3.6%)	17 (2.8%)	
Perceived Monthly Earnings situation			
Easily pass the month Pass the month without serious problems	219 (39.6%) 265 (47.9%)	245 (39.6%) 278 (45.0%)	X^2^(4, N = 1171) = 3.163, *p* = 0.5309
Trouble making ends meet in the month	43 (7.8%)	55 (8.9%)	
Barely making ends meet in the month	8 (1.4%)	16 (2.6%)	
Missing	18 (3.3%)	24 (3.9%)	
Highest level of education (ISCED 97 classifications)			
1 (Primary or lower education) 2 (Lower secondary education)	30 (5.4%) 53 (9.6%)	33 (5.3%) 65 (10.5%)	X^2^(5, N = 1171) = 1.348, *p* = 0.9299
3 (Upper secondary education)	142 (25.7%)	165 (26.7%)	
4 (Bachelor’s degree)	68 (12.3%)	83 (13.4%)	
5 & 6(Master’s or Doctoral degree)	248 (44.8%)	261 (42.2%)	
Missing	12 (2.2%)	11 (1.8%)	

^a^Age in years at the day of measurement.

^b^Body Mass Index is equal to the weight in kilograms divided by the height in meters squared as measured during the measurement date.

^c^The Waist-Hip Ratio is equal to the ratio between the waist and hip circumference in cm as measured during the measurement date.

## Discussion

In this study, we tested whether online access to multiple language versions of a German invitation letter increased active responses and eventual participation of students from non-German speaking households to a population-based health study. Using a hybrid physical-digital approach, potential participants received either the physical invitation material in German alone or together with an additional flyer, in the form of a sticky note attached to the first page, containing a QR code and instructions for accessing digital translations.

Our results demonstrate that non-German speaking households benefited when the invitation included the flyer. While adding the flyer did not increase the odds of active responses (i.e. returned consent forms), it did increase the odds of participation in the anthropometric measurements by about 15%. Both the odds of active responses and study participation were much lower in low-SSL schools, while the odds of participation were markedly elevated in high-SSL schools, replicating well established findings that socioeconomic status has an impact on involvement in health research [e.g.,^18,39^].

Exploring the interaction between the flyer, SSL, and percentage of non-German speaking households revealed that the flyer indeed had a differential effect depending on the social setting. In specific, the ROR between the flyer and the control group for both active responses and study participation rose by more than one percent per percentage point increase of non-German speaking households in classrooms in a medium-SSL. Thus, as the percentage of non-German speaking households increased, the benefit of the flyer became more advantageous. No such relationship was observed in classrooms in low- and high-SSL. However, in the control group, the odds of active responses and participation in classrooms in low-SSL were significantly lower than those in medium-SSL. Notably, although suggestive, the absence of similar differences in the flyer group does not provide evidence of a difference between the flyer and control group in that regard.

The observed pattern of results may indicate that our hybrid approach was not as effective among students from schools with low-SSL, suggesting that it might not reach the most vulnerable households with equal significance. Indeed, language barriers are only one of several barriers (e.g., digital literacy, accessibility, etc.) that disproportionately affect groups with lower socio-economic status, thereby reducing their participation in research studies^40,41^. In schools with high SSL our hybrid approach was less effective as well, suggesting that language was less of a barrier. These schools, however, had the highest proportions of engagement as well as the lowest proportion of CALD students, which may have limited the effect of a flyer aiming at language deficiencies. Another possible explanation is that parents from non-German speaking households in higher SSL may already have the resources to bridge their own language gaps. For instance, they might have better access to support networks, or be more accustomed to using other digital resources (e.g., MT apps). Furthermore, the reason to maintain a non-German household language may be different in settings with higher educational attainment: parents may speak the German language well, but still prefer non-German languages at home because they want to provide their children with opportunities to learn additional languages (e.g., their own native language).

Since only 35 of the 74 invited schools participated in this study (response proportion of 47%), it is reasonable to question whether selection effects at the school level might have biased our results, possibly limiting the external validity of the results. For instance, it might be possible that the sample consisted primarily of schools predisposed to engage in research, which might differ from nonparticipating schools in characteristics relevant to our study. However, repeating the analyses with survey weights correcting for differences in the distribution of these variables in the sample and among all schools in Bremen replicated the results from the unweighted analyses, with the notable exception of a stronger overall main effect of the flyer on active responses. While finding supports the generalizability of the results of the study at the level of schools, similar selection effects might be relevant at the individual level: participating students and their households may differ systematically from those who did not participate. Since no information is available about non-responders, this concern cannot be ruled out for the current study. However, this is the fundamental challenge for nonresponse research in general: not much (or even nothing) is known of the people that do not participate.

In a further analysis, we examined whether the flyer impacted the characteristics reported of students returning completed questionnaires, such as their main language spoken at home and their country of birth. The results did not point to any systematic differences between students in the flyer and control group. Since only translations of the study invitation, but not of the questionnaire, were provided, it is understandable that no differences were found between the flyer and control groups. In this respect, the COSI study provided a unique opportunity to test a physical-digital multilingual outreach approach. Conducting population-based studies in multiple languages usually requires validated translations of instruments, as well as field staff fluent in these languages. In the COSI study, however, parents were contacted only to complete the consent forms and questionnaire and not expected to interact in the in-person steps of the study. The measurements were taken with students in public schools who typically speak German well enough so that the field staff was not required to possess additional language skills nor to bring translators.

The use of multilingual hybrid strategies, such as the flyer, needs further research to empirically ascertain its potential in different settings and in consideration of further characteristics that possibly influence the participation of particular CALD subgroups in population-based initiatives^42^. At present, to the best of our knowledge, existing research is limited to one similar trial tested the use of translations for information about mammogram services in Norway^8^, while only one systematic review specific to cardiology settings reported a few advantageous use cases of QR codes for supplementing information in health-related settings^43^. Indeed, more research could also be conducted to evaluate the integration of physical and digital modes of communication, that is, the use of so-called *phygital* strategies^44^, into public health research to achieve equitable participatory recruitment methods^11,42^. The lack of evidence on the effectiveness of such strategies in different cultural, linguistic, or technological contexts, and in consideration of other predictors (e.g. SES), invites caution when generalizing existing studies outside of their study populations^16^.

Further development of similar intervention designs could integrate other elements to improve PHE outreach and recruitment. For example, an enhanced solution could incorporate a digital structure to identify potential language needs in advance (e.g., adding a poll, counting clicks per language) and MT for real-time translations into virtually any language. However, using MT in the context of scientific research and participant interaction would require a systematic evaluation of the quality of translation for legal and ethical material, which is not currently available^17^.

### Strengths and limitations

A strength of this study is the availability of school- and grade- specific information on the percentage of households where German is not the main language. In addition, the study includes a measure of each school’s social level that accounted not only for the small-scale socioeconomic characteristics of schools’ catchment areas, but also for socio-economic and cultural characteristics of their students and their families. This information allowed for an investigation into the differential effects of socioeconomic settings and language skills on the effectiveness of the flyer. Such analyses are often not possible in nonresponse research because an intrinsic characteristic of this line of research is the lack of knowledge about the individuals who do not respond. Another strength of the study is the availability of survey weights that allowed to rule out that the observed effects might be biased by selection effects at the school level.

The study had three main limitations. First, the experimental variable measured only whether the flyer was distributed to students, but there was no information on whether it was actually received or read by parents, whether the QR code was scanned, or how parents may have interacted with the website. Second, no information was available on the extent to which teachers encouraged their students to participate in the study or not. In this sense, the influence of teachers in promoting the study in particular classrooms was not measured. Third, the German COSI sub-survey had to be stopped before reaching the goal of recruiting 50 schools and, hence, this study as well. Given that the intervention already focused only on a subgroup of invited students (i.e., non-German speaking households), reducing the sample further hindered interpretation of the results.

## Conclusions

Although the effect size remained modest, our results demonstrate that adding the flyer to enable online translations of study invitations increased the odds of participation among students from linguistically diverse households in the COSI study. The observed effect was mainly driven by schools in medium-SSL, where the flyer positively influenced participation and active responses. These results mark a meaningful step toward increasing participation in the COSI study through a simple physical-digital multilingual strategy. However, the inconclusive results in low- and high-SSL indicate that such solutions are not easily generalized across different socioeconomic settings. Furthermore, the results suggest that language is only one of several barriers to participation in population-based research. Hybrid outreach efforts for CALD subgroups should also consider factors beyond language and the specific context of target populations.

## Electronic supplementary material

Below is the link to the electronic supplementary material.

Supplementary Material 1

## Data Availability

Data analyzed for the current study are not publicly available due to privacy concerns, but will be made available upon reasonable request to the corresponding author (rach@leibniz-bips.de).

## Abbreviations

ATE: Average treatment effects
CALD: Culturally and linguistically diverse
CI: Confidence interval
COSI: Childhood obesity surveillance initiative
COVID-19: Corona virus disease of 2019
IQHB: Institute for Quality Development in the State of Bremen
MT: Machine translation
OR: Odds ratio
PHE: Public health and epidemiological
QR code: Quick response code
ROR: Ratio of odds ratios
SSL: Social school level
URL: Uniform resource locator
WHO: World Health Organization
