# Supplementary material for: Access to translated invitations online increases involvement of linguistically diverse households in a population-based study: a cluster randomized controlled study
Source: Sci Rep. 2025 Jul 30;15:27829. doi: 10.1038/s41598-025-12092-6 (PMC12311149; doi:10.1038/s41598-025-12092-6)
Supplement: Supplementary file 1 — Supplementary Material 1 [file 41598_2025_12092_MOESM1_ESM.pdf]

Supplementary Information for "Access to translated invitations online increases involvement of linguistically diverse households in a population-based study: a cluster randomized controlled study"

Paula S. Herrera-Espejel[1,2], Thomas Mildner[3], Hermann Pohlabeln[4], Christine Genedl[5], Lutz Jasker[5] and Stefan Rach[1,2*]

[1] Leibniz Institute for Prevention Research and Epidemiology - BIPS, Department of Epidemiological Methods and Etiological Research, Bremen, Germany.
[2] Leibniz ScienceCampus Digital Public Health, Bremen, Germany.
[3] Digital Media Lab, University of Bremen, Bremen, Germany.
[4] Leibniz Institute for Prevention Research and Epidemiology - BIPS, Department of Biometry and Data Management, Bremen, Germany.
[5] Institute for Quality Development in the State of Bremen (IQHB), Bremen, Germany.

*Correspondence to:
Dr. Stefan Rach
Leibniz Institute for Prevention Research and Epidemiology - BIPS
Achterstr. 30, 28359 Bremen, Germany
rach@leibniz-bips.de

Contents

Supplementary Questionnaire Q1: Questionnaire on the quality of machine translated versions of study invitation material

Supplementary Equation E1: Multilevel mixed regression model for the primary analysis (Model 1)

Supplementary Table T1: Illustration of the conditions that are estimated in the three-way interaction (Flyer x SSL x Non-German households) in Models 1 and 2.

Supplementary Table S2: Weighted Mixed Effects Logistic Regression Model for outcome "Active response of household to study invitation" (i.e., Returned Consent Forms) for all invited students (n=4582) and "Study Participation" for all eligible students (n=4557)

Supplementary Table S3: Average treatment effects (ATE) and 95% CIs calculated from unweighted and weighted Mixed Effects Logistic Regression Models for outcome "Active response of household to study invitation" (Model 1) and "Study participation" (Model 2)

Supplementary Table S4: Weighted Mixed Effects Logistic Regression Models with interactions for outcome "Active response of household to study invitation" (i.e., Returned Consent Forms) for all invited households (n=4582) and "Study Participation" for all eligible students (n=4557) (incl. covariates)

Supplementary Questionnaire Q1: Questionnaire on the quality of machine translated versions of study invitation material

QUESTIONNAIRE [*Language*]

1. Does the disclosure notice (text in red at the top of the document) make it clear that the text was translated automatically using Google Translate services and therefore might contain grammatical or style errors?
    - YES
    - NO

2. Does the text make it clear that this activity is part of a health survey by the World Health Organization (WHO)?
    - YES
    - NO

3. Does the translated content make it clear that both, girls and boys, are invited to participate in the study?
    - YES
    - NO

4. Does the text make it clear that participation in the WHO COSI study is voluntary and that one will not incur any disadvantages if they do not want to participate?
    - YES
    - NO

5. Does the text make it clear that the study consists of collecting data on body measurements of the children (height, weight and circumference of hip and waist)?
    - YES
    - NO

6. Does the text make it clear that children cannot participate without parents filling out the declaration of consent within one week, and giving it back to the children to hand in at their school?
    - YES
    - NO

7. Does the text make it clear that the study parents are invited to answer a questionnaire on nutrition and exercise, as well as their nationality and other family aspects?
    - YES
    - NO

8. Does the text make it clear that children can take part even if parents do not fill the questionnaire?
    - YES
    - NO

9. Does the text make it clear that all legal data protection requirements are observed in this study?
    - YES
    - NO

10. If you have any specific remarks or comments you would like to share with us, we are more than appreciative to read them.

Supplementary Equation S1: Multilevel mixed regression model for the primary analysis (Model 1)

$$\log\left[\frac{\mathrm{P}(consentReturned\ =\ yes)}{1-\mathrm{P}(consentReturned\ =\ yes)}\right]$$
$$= \alpha_{j[i],k[i]} + \beta_1(flyer_{yes}) + \beta_2(perc\_nonGER) + \beta_3(grade_3) + \beta_4(perc\_female) + \beta_5(nStudentsClass) + \beta_6(flyer_{yes} \times perc\_nonGER)$$

$$\alpha_j \sim N\Big(\gamma_0^{\alpha} + \gamma_1^{\alpha}(SSL_{low}) + \gamma_2^{\alpha}(SSL_{high}) + \gamma_3^{\alpha}(nStudentsSchool) + \gamma_4^{\alpha}(flyer_{yes} \times SSL_{low}) + \gamma_5^{\alpha}(flyer_{yes} \times SSL_{high}) + \gamma_6^{\alpha}(perc\_nonGER \times SSL_{low}) + \gamma_7^{\alpha}(perc\_nonGER \times SSL_{high}) + \gamma_8^{\alpha}(flyer_{yes} \times perc\_nonGER \times SSL_{low}) + \gamma_9^{\alpha}(flyer_{yes} \times perc\_nonGER \times SSL_{high}), \sigma_{\alpha_j}^2\Big)$$, for school:district j = 1, ... ,J

$$\alpha_k \sim N(\mu_{\alpha_k}, \sigma_{\alpha_k}^2)$$, for district k = 1, ... ,K

**Variable**	**Explanation**
consentReturned	consent form returned (yes vs. no)
flyer	assignment to flyer group (yes vs. no)
perc_nonGER	percentage of students from non-German speaking households in grade (grand mean centered)
grade	grade (two or three)
perc_female	percentage of female students in classroom (grand mean centered)
SSL	social school level (low vs. medium vs. high), dummy coded with reference level “medium”
nStudentsSchool	number of students in school
nStudentsClass	number of Students in classroom
school	unique school identifier, nested in district
district	unique identifier for school districts

Supplementary Table S1: Illustration of the conditions that are estimated in the three-way interaction (Flyer x SSL x Non-German households) in Models 1 and 2.

	Estimated condition		
	flyer	SSL	Non-German households
Flyer			
No (Control)	no	Medium	0[a]
Yes	yes	Medium	0 [a]
Non-German households			
Mean (SD)	no	Medium	pct_nonGER
SSL			
Low	no	Low	0 [a]
Medium	no	Medium	0 [a]
High	no	High	0 [a]
Flyer x Non-German households			
Mean (SD)	yes	Medium	pct_nonGER
Flyer x SSL			
Low	yes	Low	0 [a]
Medium	yes	Medium	0 [a]
High	yes	High	0 [a]
SSL x Non-German households			
Low	no	Low	pct_nonGER
Medium	no	Medium	pct_nonGER
High	no	High	pct_nonGER
Flyer x SSL x Non-German households			
Low	yes	Low	pct_nonGER
Medium	yes	Medium	pct_nonGER
High	yes	High	pct_nonGER

[a] Mean of pct_nonGER, which is 0 because the variable is grand mean centered.

Supplementary Table S2: Weighted Mixed Effects Logistic Regression Model for outcome "Active response of household to study invitation" (i.e., Returned Consent Forms) for all invited students (n=4582) and "Study Participation" for all eligible students (n=4557)

	Model 1: Active Response of Invited Households (n = 4582)				Model 2: Study Participation of Eligible Participants (n = 4557)			
	No (N=2383) n (%)	Yes (N=2199) n (%)	OR	95% CI	No (N=3346) n (%)	Yes (N=1211) n (%)	OR	95% CI
Main Variables								
Flyer								
No (Control)	1198 (50.3)	1078 (49.0)	1	reference	1680 (50.2)	571 (47.2)	1	reference
Yes	1185 (49.7)	1121 (51.0)	**1.147**	1.015 – 1.297	1666 (49.8)	640 (52.8)	**1.161**	1.014 – 1.331
Non-German households								
Mean (SD)	2.40 (22.2)	-2.60 (21.1)	1.002	0.994 – 1.010	1.33 (21.8)	-3.67 (21.1)	1.002	0.994 – 1.009
SSL								
Low	848 (35.6)	440 (20.0)	**0.417**	0.272 – 0.641	1062 (31.7)	226 (18.7)	**0.582**	0.399 – 0.848
Medium	952 (39.9)	940 (42.7)	1	reference	1408 (42.1)	484 (40.0)	1	reference
High	583 (24.5)	819 (37.2)	1.278	0.832 – 1.962	876 (26.2)	501 (41.4)	**1.656**	1.213 – 2.262
Covariates								
School Grade								
Second	1202 (50.4)	1141 (51.9)	1	reference	1693 (50.6)	625 (51.6)	1	reference
Third	1181 (49.6)	1058 (48.1)	0.901	0.794 – 1.023	1653 (49.4)	586 (48.4)	0.888	0.772 – 1.021
Classroom Size								
Mean (SD)	-0.255 (3.54)	0.277 (3.49)	1.039	0.998 – 1.083	-0.119 (3.57)	0.329 (3.36)	0.993	0.955 – 1.032
Percentage of Female								
Mean (SD)	-0.350 (8.27)	0.379 (8.07)	0.994	0.985 – 1.003	-0.103 (8.22)	0.285 (8.06)	0.993	0.983 – 1.003
School Size								
Mean (SD)	1.55 (36.3)	-1.68 (35.6)	0.996	0.991 – 1.002	0.609 (36.1)	-1.68 (35.6)	1.000	0.996 – 1.005

Supplementary Table S3: Average treatment effects (ATE) and 95% CIs calculated from unweighted and weighted Mixed Effects Logistic Regression Models for outcome "Active response of household to study invitation" (Model 1) and "Study participation" (Model 2)

	Model 1: Active Response of Invited Households	Model 2: Study Participation of Eligible Participants
	ATE (95% CI)	ATE (95% CI)
Unweighted analysis	1.85 (-1.05 - 4.74)	2.61 (0.06 – 5.16)
Weighted analysis	3.24 (0.04 – 6.12)	2.84 (0.03 – 5.43)

Supplementary Table S4: Weighted Mixed Effects Logistic Regression Models with interactions for outcome "Active response of household to study invitation" (i.e., Returned Consent Forms) for all invited households (n=4582) and "Study Participation" for all eligible students (n=4557) (incl. covariates)

	Model 1a: Active Response of Invited Households (n = 4582)				Model 2a: Study Participation of Eligible Participants (n = 4557)			
	No (N=2383) n (%)	Yes (N=2199) n (%)	OR	95% CI	No (N=3346) n (%)	Yes (N=1211) n (%)	OR	95% CI
Main Variables								
Flyer								
No (Control)	1198 (50.3)	1078 (49.0)	1	reference	1680 (50.2)	571 (47.2)	1	reference
Yes	1185 (49.7)	1121 (51.0)	1.014	0.819 – 1.256	1666 (49.8)	640 (52.8)	1.009	0.794 – 1.283
Non-German households								
Mean (SD)[b]	2.40 (22.2)	-2.60 (21.1)	0.997	0.983 – 1.012	1.33 (21.8)	-3.67 (21.1)	0.999	0.984 – 1.014
SSL								
Low	848 (35.6)	440 (20.0)	**0.355**	0.210 – 0.603	1062 (31.7)	226 (18.7)	**0.462**	0.273 – 0.782
Medium	952 (39.9)	940 (42.7)	1	reference	1408 (42.1)	484 (40.0)	1	Reference
High	583 (24.5)	819 (37.2)	1.181	0.643 – 2.170	876 (26.2%)	501 (41.4)	**1.774**	1.019 – 3.088
Two-way Interactions								
Flyer x Non-German households								
Mean (SD)	1.88 (21.8)	-0.747 (20.8)	1.014	1.000 – 1.029	1.45 (21.5)	-2.31 (21.0)	**1.019**	**1.002 – 1.035**
Flyer x SSL								
Low	416 (35.1)	247 (22.0)	1.464	0.952 – 2.251	533 (32.0)	130 (20.3)	1.617	0.957 – 2.733
Medium	476 (40.2)	485 (43.3)	1	reference	708 (42.5)	253 (39.5)	1	Reference
High	293 (24.7)	389 (34.7)	1.164	0.688 – 1.971	425 (25.5)	257 (40.2)	0.902	0.522 – 1.559
SSL x Non-German households								
Low	18.7 (21.4)	19.2 (18.9)	1.002	0.981 – 1.023	18.5 (20.9)	19.5 (19.0)	1.001	0.978 – 1.024
Medium	1.44 (15.7)	3.49 (15.2)	1	reference	1.80 (15.6)	3.59 (15.1)	1	Reference
High	-19.8 (8.41)	-21.3 (9.66)	0.997	0.967 – 1.027	-20.3 (8.61)	-21.1 (9.68)	1.005	0.977 – 1.034
Three-way Interaction								
Flyer x SSL x Non-German households								
Low	18.1 (21.7)	19.3 (19.0)	0.999	0.970 – 1.028	18.2 (21.0)	18.7 (19.5)	0.979	0.957 – 1.002
Medium	0.679 (15.3)	4.68 (14.4)	1	reference	1.41 (15.2)	5.52 (13.9)	1	Reference
High	-19.2 (8.20)	-20.2 (9.56)	0.987	0.968 – 1.007	-19.6 (8.54)	-20.6 (9.71)	0.974	0.945 – 1.004
Covariates								
School Grade								
Second	1202 (50.4)	1141 (51.9)	1	reference	1693 (50.6)	625 (51.6)	1	reference
Third	1181 (49.6)	1058 (48.1)	0.912	0.802 – 1.038	1653 (49.4)	586 (48.4)	0.901	0.781 – 1.039
Classroom Size								
Mean (SD)[b]	-0.255 (3.54)	0.277 (3.49)	1.030	0.988 – 1.074	-0.119 (3.57)	0.329 (3.36)	0.985	0.946 – 1.025
Percentage of Female								
Mean (SD)[b]	-0.350 (8.27)	0.379 (8.07)	0.995	0.986 – 1.005	-0.103 (8.22)	0.285 (8.06)	0.993	0.983 – 1.003
School Size								
Mean (SD)[b]	1.55 (36.3)	-1.68 (35.6)	0.997	0.992 – 1.002	0.609 (36.1)	-1.68 (35.6)	1.000	0.995 – 1.005
